# Just let them play? Deliberate preparation as the most appropriate foundation for lifelong physical activity

**DOI:** 10.3389/fpsyg.2015.01548

**Published:** 2015-10-08

**Authors:** Áine MacNamara, Dave Collins, Susan Giblin

**Affiliations:** College of Health and Wellbeing, Institute of Coaching and Performance, University of Central LancashirePreston, UK

**Keywords:** physical activity, movement preparation, children, physical literacy, motor skills

## Introduction

Recent research in the UK suggests that nearly half of children leave school without the basic movement skills required to engage successfully in sport and physical activity (Griffiths et al., 2013). A key question therefore is how early experiences in sport and physical activity should be structured in order to provide young people with the foundation for life-long physical activity participation. Perhaps as a backlash to the demands placed on young children in (some) organized sport (Hancock et al., 2013), and reflective of the dreading of the “disappearance of childhood” (Postman, 1994), there is a growing advocacy for a play approach, with an emphasis on psychosocial rather than psychomotor development, to dominate early years participation. A common concern amongst adults is that children no longer play the way previous generations did and this, perhaps nostalgic, observation has led to calls for opportunities for children to engage in spontaneous and self-directed play. Unlike coach-led approaches aimed at instruction and the transmission of knowledge, “play curricula” are seen as child-centered and developmentally appropriate. However, the extent to which this foundation provides an effective basis for prolonged engagement in sport and physical activity is notably unsupported. The play approach appears to be built on a general presumption that movement skills and physical literacy develop naturally as a consequence of age, maturation, general movement experiences and self-discovery. However, a substantial body of research (e.g., Stodden et al., 2008; Robinson and Goodway, 2009; Giblin et al., 2014a,b) highlights how structured instruction and feedback are required to ensure that essential movement skills (EMS) develop appropriately, particularly during early childhood. Importantly, EMS incorporates not just the actual competence to perform physical skills but also the psychological and behavioral skills to engage in physical activity. Notably, the interaction between actual competence and perceived competence predicts future engagement in physical activity more accurately than either alone (e.g., Barnett et al., 2008). In this opinion piece, we argue that quality early physical experiences, delivered in an appropriate manner, are necessary to ensure the optimal development of those EMS that allow children to develop competence (both actual and perceived) in their ability to progress and execute a range of complex and combined movement skills; in short, the ability and confidence “to have a go” (Giblin et al., 2014a). A focus on the quality of this experience, rather than an, in our opinion, misplaced emphasis on (ill-defined) play experiences should be the focus of early interventions.

Deliberate play, described as activities engaged in during childhood that are inherently enjoyable and different from organized sport and adult-led practices, is promoted as an important precursor for long-term engagement in sport and physical activity (cf. Côté, 1999). We would argue however, the deliberate play paradigm is often misinterpreted and that allowing children to play without appropriate feedback, instruction or organization is unlikely to result in the learning (i.e., actual and perceived competence) required to ensure prolonged engagement; children need to be supported, guided, and encouraged through a range of developmentally appropriate tasks to facilitate acquisition of, and confidence in, the skills needed for proactive and enthusiastic participation. Furthermore, the contention (Côté and Hancock, 2014) that deliberate play can make unique contributions to skill development through implicit learning certainly requires more evidence before it can be adopted with any certainty. Although children's EMS develop with age, it is important that versatile skill practice situations are provided to promote and reinforce these skills. Specifically, it is far from proven that skill levels *naturally* reach the levels which encourage or facilitate participation (Giblin et al., 2014b). In the same way that children need to learn the ABCs before learning how to read and write, they need to learn EMS before they can become skillful and confident in playing sports and other physical activities (Goodway and Savage, 2001). Acquiring and refining EMS during early childhood enables children to engage in physical activity with competence and confidence (Goodway et al., 2009; Giblin et al., 2014a)—the essential precursors for long-term engagement. Indeed, the relationship between proficient motor skills and physical activity has been demonstrated in several cross-sectional studies (Stodden et al., 2008; Lubans et al., 2010). As such, there is considerable evidence to support the teaching of EMS through age-appropriate activities within a sequential curriculum. Like reading, writing, and maths, EMS experiences need to be planned, taught, learned, reinforced, and assessed (Robinson and Goodway, 2009). This will inevitably employ some fun, as with the teaching of anything at this age, but the benefits are unlikely to spontaneously occur in an unstructured play environment. Therefore, the importance of a supportive, learning environment that fosters these essential precursors of long-term engagement is crucial.

This is also more than just time accumulated. Significant research suggests that time spent in physical activity alone is not enough to generate positive changes in children's EMS (Fisher et al., 2005). Instead, skill specific experiences are needed although careful consideration of the appropriateness of this experience is the vital element. The extant literature suggests that the sensitive learning period for the development of EMS is between two and seven years of age (Gallahue and Cleland-Donnelly, 2007). Reflecting this, we argue that providing young children with a broad experience in a supportive environment gives them the best chance to be become successful movers—as such, it is important to recognize that children do not acquire these skills automatically as a result of the maturation process but instead it is facilitated through instruction and practice (e.g., Martin et al., 2009). EMS cannot be expected to naturally “emerge” during early childhood, at least to the level of competence needed for them to act as building blocks for later engagement, whether the individual is focused on participation or higher level performance (cf. Collins et al., 2012). Recent research (Belanger et al., 2015) suggests that type and consistency of participation during childhood is related to adult physical activity participation. Specifically, prolonged participation in organized team sports and running during childhood was shown to positively correlated to adult physical activity participation with no relationship apparent between fitness/dance activities during childhood and adult physical activity participation. Although speculative, it may be that participation in organized sports and running equipped young people with the EMS required to maintain participation through adult years, whereas the “daily dose” and participation motives associated with fitness and dance activities may not have the same long-lasting effects. The need for further research notwithstanding it seems likely that without appropriate foundations in organized sport, many children will not attain sufficient competence in EMS to be motorically competent as adults.

Clearly, the question that must be addressed is how to ensure the optimum development of EMS. For example, environmental considerations, such as the equipment used, previous experience, and instruction, may influence motor development with EMS proposed to emerge within a dynamic system consisting of a specific task, performed by a learner with given characteristics, in a particular environment. As such, a range of factors interact with the learner to influence motor skill development. A number of studies (e.g., Hamilton et al., 1999) have found that disadvantaged children demonstrated developmental delays in EMS, suggesting that these delays indicated the lack of environmental support in which the children were raised and further questioning the automatic growth assumption espoused by many of the “let them play” camp. Given these data, it is important to examine the role of intervention programs, including quality physical education and sport instruction during early childhood, in ensuring the development of EMS across populations. Notably, a range of empirical evidence supports this approach with Kelly et al. (1989), amongst others (Hamilton et al., 1999), reporting that typical preschool children demonstrated qualitative performance gains in six fundamental motor skills from pre-test to post-test as a result of two 5-week instructional units consisting of direct instruction. In contrast, the control group, who engaged in well-equipped free play, made no significant gains in motor skill development. The solution seems obvious; for students to learn the EMS required for long-term engagement in physical activity, quality interventions using effective instruction must be implemented (Graham et al., 2001). Of course, there is a need to develop standardized measurement tools to provide empirical evidence to support this statement. However, the key consideration is that young children demonstrate various levels of motor skill competence primarily because of differences in experience. These differences are the result of many factors including immediate environment, presence of structured physical education, socioeconomic status, parental influences, climate, etc. Consideration, and exploitation, of these factors within well-structured and appropriately delivered educational systems should ensure that all (or at least as many as possible) are equipped with the skills needed to maintain their involvement in physical activity and sport, especially as these perceptions of competence play an increasingly important role in adolescence. This is an important consideration because, although there is evidence showing that 4–7-year-old children's fundamental movement skills and physical activity are only weakly interrelated (Raudsepp and Pall, 2006), studies have shown that childhood motor skill proficiency influences adolescent physical activity and fitness (Barnett et al., 2008). Therefore, the ability to perform a variety of EMS, and the confidence in this ability, effectively increases the likelihood of children's participation in different physical activities throughout their lives (Haywood and Getchell, 2009).

Unfortunately, policy formulation, particularly in childhood physical activity promotion, has to date been predominated by two disparate perspectives. The first, psychosocially focused idea (deliberate play; Côté and Hancock, 2014) suggests a distinct focus on developing the psycho-social facets (fun, enjoyment, play) almost exclusively, with little guidance derived from neuroscience or motor development theory. Whilst the value of developing intrinsic motivation for being physically active is not in question, we argue that the sole focus on the psychosocial factors of physical development when unaccompanied by a sufficient level of physical skill learning is a limited approach. For example, play models suggest that unstructured activities are optimal for increasing activity and engagement during early childhood- however, for children lacking basic movement competence the experience of playing can frequently result in frustration or failure. Although self-exploration and internally generated feedback is an important part of skill acquisition, from a motor-learning stance even self-exploration, solution generation, and feedback interpretation requires careful curricular design and delivery consideration (even if not directly provided by adults) to ensure that appropriate movement information is acquired (cf. Posner and Snyder, 1975) or even to experience the positive psychosocial benefits typically associated with play (cf. Kennedy-Behr et al., 2014). More specifically, whilst basic movements emerge before the age of four surely the aim of physical education and youth sport should be to provide more advanced physical skill learning (i.e., object manipulation, interceptive timing, spatial awareness, rhythm, and sequencing). In the absence of sufficient procedural knowledge during learning phases, the level, progression and adaptation of movement skills is likely to be impeded.

The second, physiological or fitness perspective places an increasing emphasis on the fitness levels of children, presumably with the assumption that greater fitness at young ages will in some way translate into a lifelong fitness habit (cf. UK Active's Generation Inactive report, UK Active, 2015). Once again, but equally concerning, this premise is promoted without evidence. Perhaps a case of measurement will conquer all; unfortunately without any evidence of causation.

## Deliberate preparation—equipping for lifelong physical activity

So, what is the answer? Unstructured play in the early years is unlikely to afford sufficient opportunities to develop competence and confidence and it therefore follows that children should be provided with early experiences to develop a broad range of fundamental skills as these facilitate both successful early involvement in sport (a prerequisite for prolonged engagement), as well as subsequent development either at elite levels or for personal accomplishment and progression (Collins et al., 2012). Accordingly, we propose that lifelong participation in physical activity can derive from a robust foundation of psychomotor skills and that, for students to learn these skills, quality programs using effective instruction must be provided (Graham et al., 2001). This approach to physical activity promotion is called “Deliberate Preparation” (Giblin et al., 2014a). Appropriate and well-founded generic athletic skills (e.g., locomotion, balance, strength) allow flexible movement of individuals between levels and domains of PA involvement (Collins et al., 2012).

The Deliberate Preparation approach proposes that structured physical skill development during the early years could provide a situated learning environment for students to acquire the behavioral, psychological and movement skills that increase the likelihood of lifelong physical activity. Given this, and building on the relationship between enjoyment, self-determination, and perceived competence discussed previously, the conditions of children's sport involvement should focus on improving physical skill competence rather than short-term “activity quotas” or “just letting them play.” Of course, the absence of effective physical literacy assessment tools there is little data on how or if such interventions work (Bardid et al., 2015). Developing and validating such tools, and truly longitudinal work (cf. Belanger et al., 2015), is needed to support the suggestions offered in this opinion paper.

### Conflict of interest statement

The authors declare that the research was conducted in the absence of any commercial or financial relationships that could be construed as a potential conflict of interest.
